# Visibility of Surgical Site Marking After Preoperative Skin Preparation

**Published:** 2008-07-16

**Authors:** Simon C. Mears, A. Feroz Dinah, Trevor A. Knight, Frank J. Frassica, Stephen M. Belkoff

**Affiliations:** International Center for Orthopaedic Advancement, Johns Hopkins University/Johns Hopkins Bayview Medical Center, Baltimore, Maryland

## Abstract

**Objective:** It is important that during preoperative skin preparation surgical site markings are not erased. The effects of 2 common types of skin preparation solutions on surgical site markings were compared. **Methods:** Fasciocutaneous skin flaps were harvested and 20 random combinations of 3 letters were written on the skin flaps with a black permanent marker. Ten of the 3-letter combinations received Chloraprep (chlorhexidine gluconate, 2% w/v, plus isopropyl alcohol, 70% v/v) and the other 10 received Duraprep (iodine povacrylex [0.7% available iodine] and isopropyl alcohol [74% w/w]), both according to the manufacturer's guidelines. The skin flaps were photographed digitally before and after application of the solutions. The final pictures were assessed subjectively by 10 surgeons and then objectively to determine the change in visibility of the marking on each specimen. **Results:** Of the 300 letters in each group, the number of correctly identified letters was 254 (84.7%) in the Chloraprep group and 284 (94.7%) in the Duraprep group. On the basis of the visibility of skin markings, Chloraprep was 21.8 times more likely (95% credible interval, 7.3–86.7) to erase the site markings than was Duraprep. **Conclusions:** Skin preparation with Chloraprep erased more surgical site markings than did Duraprep.

Marking the surgical site is essential for the planning of any surgical procedure and for the prevention of wrong-site surgery. Recently, the American Academy of Orthopaedic Surgeons^1^ issued an advisory statement and developed the “sign your site” awareness campaign to reduce the incidence of wrong-site surgery. In 2003, the Joint Commission on Accreditation of Healthcare Organizations^2^ developed guidelines that require preoperative verification of the surgery site and patient, marking of the surgical site on the patient, and a “time-out” in the operating room to verify the patient data. In addition, several specialties use skin marking to design surgical incisions preoperatively. Mammoplasty, for example, requires the markings to be made with the patient in the supine position and anesthetized.

The Centers for Disease Control and Prevention^3^ has recommended the use of “an appropriate antiseptic agent for skin preparation” to prevent surgical site infections. The most common source of postoperative infection is the patient's own skin flora.^4^ Surgical skin preparation solutions usually contain iodine or chlorhexidine and they can be aqueous or alcoholic. Two common products used for skin preparation are Chloraprep (chlorhexidine gluconate, 2% w/v and isopropyl alcohol, 70% v/v; Enturia Inc, Leawood, Kansas) and Duraprep (Iodophor, 0.7% available iodine and isopropyl alcohol, 74% w/w; 3M Healthcare, Saint Paul, Minnesota). Chlorhexidine gluconate, a component of Chloraprep, is a chemical antiseptic that is bactericidal and fungicidal with little effect on spores, mycobacteria, and viruses. The mechanism of action is thought to be membrane disruption.^5^ Iodophor (0.7% available iodine), a component of Duraprep, is a complex of iodine and a solubilizing agent or carrier, which acts as a reservoir of the active, “free” iodine. Iodine is rapidly bactericidal, fungicidal, tuberculocidal, virucidal, and sporicidal.^5^ It is noteworthy that both surgical preparations contain 70% or more isopropyl alcohol, which exhibits rapid, broad-spectrum antimicrobial activity against vegetative bacteria (including mycobacteria), viruses, and fungi but not spores. Little is known about the specific mode of action of alcohols but it is generally thought that they cause membrane damage and rapid denaturation of proteins, with subsequent interference with metabolism and cell lysis.^5^ Evidence on which to base a choice between these products is sparse and sometimes conflicting. In terms of reducing infection, chlorhexidine gluconate has been shown to be superior to povidone iodine for skin preparation for vascular catheter insertion.^6^ Chlorhexidine gluconate also has been shown to be better than povidone iodine in reducing bacterial skin counts for procedures such as vaginal hysterectomy^7^ and foot and ankle surgery.^8^,^9^ Duraprep has been shown to be superior in allowing adhesion of drapes to skin.^10^ Despite the lack of evidence of superiority of one solution versus another, patient safety specialists are recommending that chlorhexidene solutions be used for surgical skin preparation. In our and other hospital systems, this recommendation is becoming a mandatory switch to one particular product, a chlorhexidene-based skin preparation solution for which a 30-second scrub application is recommended.

We have observed that the skin preparation often smudges the marks, rendering them illegible, or erases them completely. In our study, we compared the effects of 2 widely available surgical skin preparation solutions, Chloraprep and Duraprep, on skin markings.

## METHODS

### Specimens and procedures

Three skin flaps were harvested from the thighs of Caucasian male cadavers obtained from the State Anatomy Board. Before marking, the flaps were thawed to room temperature (20°C), as confirmed with a thermocouple (K-type, Omega Engineering Inc, Stamford, Connecticut) inserted into the epidermis.

To replicate initials typically seen in the operating room for site marking, 20 random 3-letter combinations were generated with MS Excel (Microsoft Office 2003, Microsoft Corporation, Seattle, Washington). A black permanent marker routinely used in the operating room (Super Sharpie Series 33000, Sanford Corporation, Oak Brook, Illinois) was used to mark the skin. The initials were drawn as capital letters (4- to 5-cm tall) and underlined. The writing was allowed to dry for at least 15 minutes before the solution was applied. Pre-preparation pictures then were obtained with a digital still camera (Digital Rebel XTi, Canon USA, Lake Success, New York) with a 100-mm macro lens (EF 100mm f/2.8 USM Macro Lens, Canon USA, Lake Success, New York) and ring flash (MR-14EX TTL, Canon USA, Lake Success, New York). The camera captures at 10.1 megapixels and the shutter speed was set at 1 per 60 seconds with an F-stop value of 4.0. In all cases, we used a tripod to position the camera at a fixed distance from the skin.

Half of the initials were prepped with Chloraprep and the other half with Duraprep. The solutions were applied according to the manufacturers' guidelines. For the Chloraprep, the method involved repeated forward and backward strokes for 30 seconds. For the Duraprep, the method involved painting a single layer of the solution onto the skin without scrubbing it. All specimens were allowed to dry fully for at least 3 minutes. Postpreparation pictures were taken, numbered randomly, and made into a digital presentation (PowerPoint, Microsoft Office 2003, Microsoft Corporation, Seattle, Washington). The presentation was shown separately to 10 surgeons. Each was asked to write down the letters in each photograph and to indicate whether the initials could be seen well enough to perform a surgical time-out.

All raw digital pictures were converted into a gray scale with 256 levels, using Adobe Photoshop CS2 (Adobe, San Jose, California), with 0 being the darkest and 255 being white. The histogram tool was used to determine the mean gray level of the line drawn under each set of initials (for quantitative assessment) and of the strip of adjacent skin. The difference between the 2 mean gray levels provided a measure of the contrast between the writing and the skin in any given image. This contrast was determined for each specimen in the preapplication and postapplication photographs.

The effect of skin preparation on the odds of recognizing skin markings was analyzed using the Bayes method with a normal (0, 10E6) prior distribution (WinBUGS, Version 1.4, Imperial College, London, UK).

## RESULTS

### Subjective assessment

**Figure 1a–1d:**
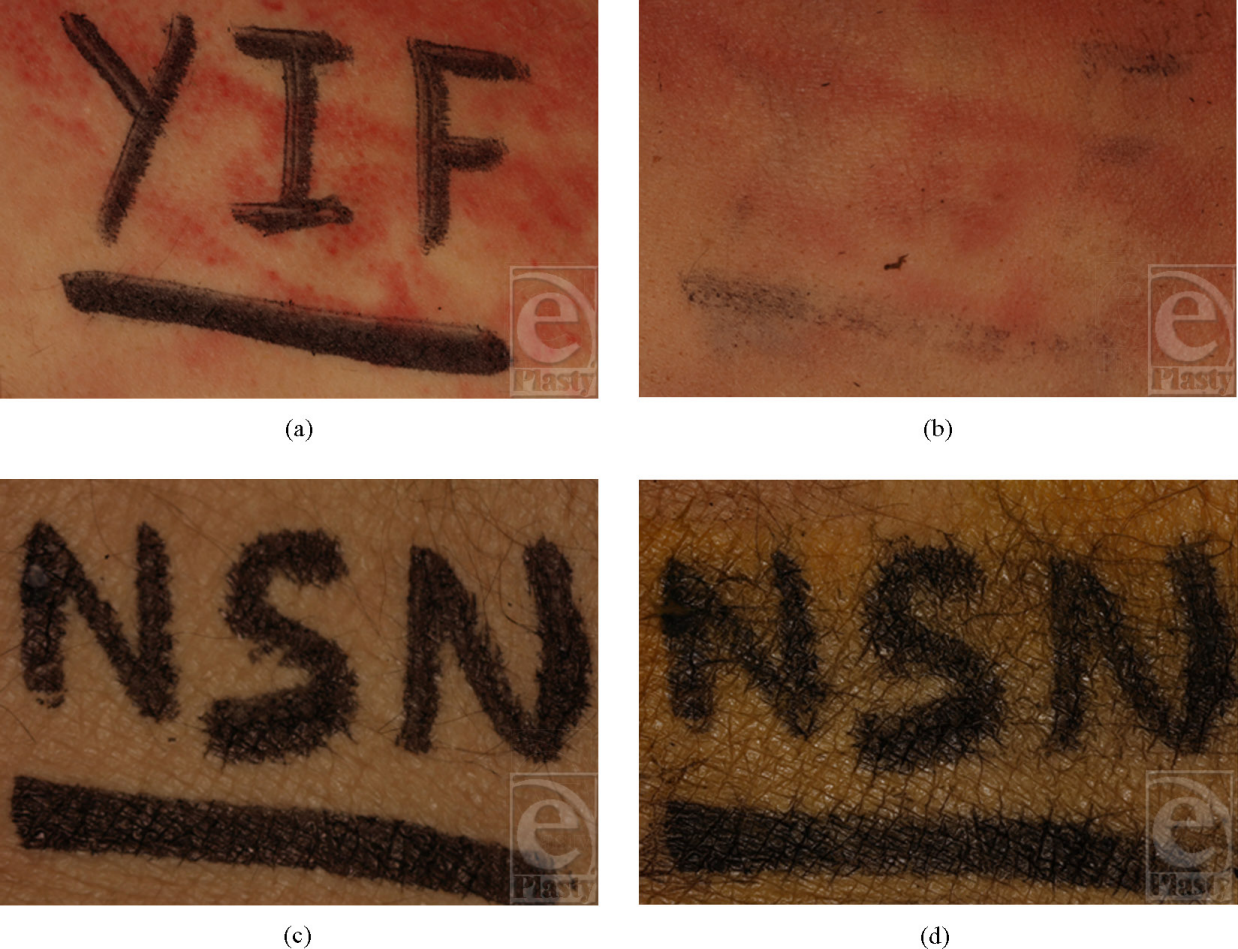
Specimens before and after skin preparation with a chlorhexidine-based solution (a and b, respectively) or an iodine-based solution (c and d, respectively).

The 10 surgeons correctly identified 254 of 300 individual letters in the Chloraprep group and 284 of 300 letters in the Duraprep group (84.7% vs 94.7%, respectively). Only 58 of 100 sets of initials (58%) in the Chloraprep group were recognized well enough (Fig 1) by the surgeon to perform a surgical time-out, compared with 92 of 100 sets (92%) in the Duraprep group. The posterior distribution of odds ratios indicated that Chloraprep was 21.8 times more likely (95% credible interval, 7.3–86.7) to erase the initials than Duraprep.

### Objective assessment

The mean change in gray scale contrast for the Chloraprep group was 39.3 units (95% credible interval, 30.8–48.3), whereas that for the Duraprep group was 17.7 units (95% credible interval, 9.4–26.1). This change in contrast indicates that the marks in the Chloraprep group were erased more than the marks in the Duraprep group.

## DISCUSSION

In our study, we showed that skin preparation with Chloraprep was more likely to erase site markings than skin preparation with Duraprep. With increasing awareness about the prevention of wrong-site surgery, visibility of the site marking is essential. Indeed, one of the recommendations of the Joint Commission on Accreditation of Healthcare Organizations^2^ is that “the intended site must be marked such that the mark will be visible after the patient has been prepped and draped.” Site-marking erasure creates a major problem for the surgeon and operating personnel. During plastic surgery, these markings, essential for the reconstructive plan, are made with the patient awake and cannot be recreated with the patient anesthetized. The erasure of site identification markings also creates a problem in performing an accurate surgical time-out. One solution used in some hospitals is to do the time-out before skin preparation. In our institution, hospital policy indicates that surgical time-out should be done after skin preparation and draping and just before skin incision. Interestingly, the Universal Protocol of the Joint Commission on Accreditation of Healthcare Organizations^2^ provides no guidance on this matter, other than mentioning that any difference of opinion between members of the surgical team needs to be resolved before the procedure is started. The American College of Surgeons^11^ suggests that the surgical site listed on the consent form should be confirmed with the patient or the patient's designated representative. The use of a surrogate decision maker creates many practical questions and room for error. Clearly, the solution is to prevent site-marking erasure. Erasure creates a culture within the operating room setting that denigrates the importance of the surgical time-out in starting surgery, which represents a retrograde step in the knowledge-attitudes-behavior model for harm prevention.^12^

Chloraprep and Duraprep are effective and are used widely throughout the United States. Each has its advocates, based partly on studies^6^–^9^ showing the efficacy of chlorhexidine gluconate and povidone iodine. A study in foot and ankle surgery^9^ showed that a chlorhexidine-based solution was superior to the iodine-based solution according to quantitative culture specimens from various parts of the foot. In vitro experiments have shown povidone iodine to be superior to chlorhexidine gluconate in terms of killing methicillin-resistant *Staphylococcus aureus*^13^ and reducing the incidence of antimicrobial resistance.^14^

Although it is possible that removal of marks is a result of a reaction between the chlorhexidine gluconate and iodophor on the synthetic pigment present in the black marker, it seems probable that the difference seen is the result of dissimilar application of the preparations onto the skin. According to the manufacturer's guidelines, Chloraprep should be applied by repeated forward and backward strokes for 30 seconds. For Duraprep, the manufacturer recommends painting a single layer of the solution onto the skin without scrubbing it. The scrubbing action may be responsible for the greater degree of marking removal in the chlorhexidine group. Because both preparations contain approximately 70% isopropyl alcohol, that chemical solvent is unlikely to play an important role in the difference seen.

Our study has several limitations. It is possible that dermoepidermal separation seen in postmortem tissue secondary to rubbing the skin may confound our results. It was not possible to standardize the amount of ink used to mark each site. Nevertheless, the application of ink letters was consistent between specimens assigned to both preparation groups. We used a standard marking pen for this study but the particular marker we used could be more susceptible to the effects of Chloraprep than other surgical markers. In the future, the effects of skin preparation on other types of pen markings are needed to determine whether a better skin marker exists.

In summary, skin preparation with Chloraprep erased more surgical-site markings than did Duraprep. Clinical trials comparing the erasure of skin markings with these 2 solutions are needed to validate our findings.
